# Pre-Weaned Calf Rearing on Northern Irish Dairy Farms—Part 2: The Impact of Hygiene Practice on Bacterial Levels in Dairy Calf Rearing Environments

**DOI:** 10.3390/ani13061109

**Published:** 2023-03-21

**Authors:** Aaron J. Brown, Gillian Scoley, Niamh O’Connell, Alan Gordon, Katie Lawther, Sharon A. Huws, Steven J. Morrison

**Affiliations:** 1 Agri-Food and Biosciences Institute, BT26 6DR Hillsborough, Ireland; 2 Institute for Global Food Security, School of Biological Sciences, Queen’s University Belfast, BT9 5DL Belfast, Ireland; 3 Agri-Food and Biosciences Institute, Newforge Lane, BT9 5PX Belfast, Ireland

**Keywords:** bedding, enteric disease, housing, feeding equipment, management, space allowance

## Abstract

**Simple Summary:**

Good management of hygiene is required to minimise the risk of disease transmission in pre-weaned calf rearing facilities. In this study, calf rearing facilities were surveyed and samples of feed, bedding and feeding equipment were collected as they are likely sources of infection in calves. These samples were analysed for hygiene indicators including total viable count (TVC), coliforms (TCC) and *Escherichia coli* and compared with pre-defined target levels for each of these indicators. Use of automatic feeders rather than manually mixed milk replacer or cow’s milk led to an increased likelihood of the milk/milk replacer TVC being within target levels, and solid feed was less likely to contain *E. coli* when sampled from group pen buckets compared with single pens. Water samples from self-fill drinkers were more likely to contain high TVC than samples from manually filled drinkers, and single pens were more likely to have high TCC than group pens. Drinker and pen type were confounded though, with all the self-fill drinkers located in group pens. Cleaning milk feeders after every feed reduced the chance of high TVC levels, and high levels of TVC in feeders were linked to high levels in milk mixing utensils. Bedding was more likely to contain target TVC and TCC levels in group than in single pens. This may reflect differences in space allowance and floor type between group and individual pens. The results showed variation of hygiene levels across farms and highlighted several associated management and housing factors.

**Abstract:**

Pre-weaned dairy calves are very susceptible to disease in the first months of life due to having a naïve immune system and because of the numerous physiological stressors they face. Hygiene management is a key element in minimizing enteric disease risk in calves by reducing their exposure to pathogens. Samples of milk, concentrate feed and drinking water, boot swabs of bedding and swabs of feed equipment were collected from 66 dairy farms as part of a survey of calf rearing practice and housing design. All the samples were cultured to determine total viable counts (TVC), total coliforms (TCC) and *Escherichia coli* as indicators of hygiene. Target ranges for levels of TVC, TCC and *E. coli* were defined from the literature and the sample results compared against them. The TVC targets in milk, MR and water were <4.0 log_10_ CFU/mL. TCC and *E. coli* targets of <1.1 log_10_ CFU/mL (the detection limit) were used for milk, MR, concentrate feed and feeding equipment. For water, the TCC and *E. coli* targets were <1.0 log_10_ CFU/100 mL. The targets used for bedding boot swabs were <6.3 log_10_ TVC CFU/mL and <5.7 log_10_ TCC or *E. coli* CFU/mL. Farm management factors were included as fixed effects in a generalized linear mixed model to determine the probability of samples being within each hygiene indicator target range. Milk replacer samples obtained from automatic feeders were more likely to be within the TVC target range (0.63 probability) than those prepared manually (0.34) or milk samples taken from the bulk tank (0.23). Concentrate feed samples taken from buckets in single-calf pens were more likely to have *E. coli* detected (0.89) than samples taken from group pen troughs (0.97). A very small proportion of water samples were within the indicator targets (TVC 9.8%, TCC 6.0%, *E. coli* 10.2%). Water from self-fill drinkers had a lower likelihood of being within the TVC target (0.03) than manually filled buckets (0.14), and water samples from single pens were more likely to be within TCC target ranges (0.12) than those from group pens (0.03). However, all self-fill drinkers were located in group pens so these results are likely confounded. Where milk feeders were cleaned after every feed, there was a greater likelihood of being within the TVC target range (0.47, compared with 0.23 when not cleaned after every feed). Detection of coliforms in milk replacer mixing utensils was linked with reduced probability of TVC (0.17, compared with 0.43 when coliforms were not detected) and TCC (0.38, compared with 0.62), which was within target in feeders. Key factors related to increased probability of bedding samples being within TCC target range were use of group calf pens (0.96) rather than single-calf pens (0.80), use of solid floors (0.96, compared with 0.76 for permeable floors) and increased space allowance of calves (0.94 for pens with ≥2 m^2^/calf, compared with 0.79 for pens with <2 m^2^/calf). Bedding TVC was more likely to be within the target range in group (0.84) rather than in single pens (0.66). The results show that hygiene levels in the calf rearing environment vary across farms and that management and housing design impact hygiene.

## 1. Introduction

Management of health and disease is an important aspect of ensuring that dairy calves thrive in the first few months of life. Traditionally, neo-natal calves are among the highest risk animals on dairy farms, due to their naïve immunity and the numerous stressors encountered within this time period [1,2,3]. The predominant diseases causing mortality and morbidity in pre-weaned calves are enteritis in the first month and pneumonia after the first month [4]. These diseases lead to increased costs associated with veterinary treatments, reductions in feed efficiency and growth performance, and adverse effects on long-term development [5,6]. Additionally, the pain and distress caused by disease negatively impacts calf welfare [7]. Therefore, minimizing the risk and spread of disease is essential.

As the pathogens causing calf enteritis, such as *Salmonella* spp., *Cryptosporidium parvum*, rotavirus and coronavirus [4], are commonly spread by faecal–oral transmission, the environment that the calf is reared in needs to be clean. McGuirk [4] highlighted the sources of calf enteric disease causing pathogens as pen bedding and other surfaces, feed, feeding equipment and skin. Routine cleaning of the calf rearing environment is generally undertaken on farms and is an established method to reduce the exposure of calves to these pathogens [8]. The goal of cleaning, as described by Hancox et al. [9], is to remove organic matter and kill remaining micro-organisms through the use of chemical disinfection and natural desiccation (through resting the equipment or facilities once dry). However, the frequency and thoroughness of cleaning varies across farms, which likely influences the level of micro-organisms present in calf pens or feeding equipment [10]. Detergent and disinfectant use is instrumental in breaking down biofilms and removal of pathogens [9] but requires the surface to be completely free of organic debris [4]. However, recent research has indicated that some dairy farms in Northern Ireland do not use any such products when cleaning calf rearing facilities and equipment [11]. Lack of use of detergents and/or disinfectants on dairy farms has also been found in other countries [10,12,13]. In cases where organic matter is not removed or the remaining surface is not disinfected, it is likely the process will not be effective. This results in the persistence of pathogens, causing increased challenge for young calves [9]. Failing to remove pathogens from facilities and equipment may also prevent a break in the cycle of disease within a calfhouse.

The surface quality of feeding equipment and calf pens may influence the extent to which cleaning and disinfection removes organic matter and micro-organisms [8]; cracks or crevices can be inaccessible to cleaning products or tools and can, therefore, provide protection to micro-organisms. Additionally, Hancox et al. [9] found variation in the effectiveness of disinfection and rest periods when applied to different pen surface materials, with disinfection being successful on concrete and stock board (recycled plastic sheeting) but not on metal. Furthermore, the stocking density of calf pens is an important factor in hygiene, as it has been seen to greatly influence airborne bacteria levels [14]. Increased stocking density has previously been seen to increase calf morbidity [15], although increased social stress on calves due to overcrowding could also negatively impact health [16].

Similarly, feedstuffs can also be a source of bacterial challenge to young calves. For example, poor colostrum hygiene has been established as a route of pathogen transfer for enteric disease but may also reduce the absorption efficiency of important nutrients and immunoglobulins [17]. Pathogenic bacteria compete with beneficial bacteria species in the digestive tract and may alter gut health and nutrient digestion [18]. Thus, milk hygiene is considered a risk factor for disease, but provided collection and feeding equipment is clean, milk is unlikely to be heavily contaminated [4]. Surprisingly, water hygiene is rarely evaluated as a factor in faecal–oral transmission in calves despite it being a key source of disease in humans [19], poultry [20] and pigs [21]. Water hygiene has been shown to influence cattle performance on beef farms as palatability drives intake and subsequent solid feed intake [22]. Despite the importance of water in the diet of calves [23], to the authors’ knowledge, no studies have evaluated water quality in pre-weaned dairy calf operations.

As hygiene management requires time-consuming input from producers, it is important that it results in clear benefits to the cleanliness of the calf facilities and ultimately to the health and welfare of the calves. Little work has evaluated the effectiveness of various hygiene protocols for calf pens and feeding equipment. In order to address this, a survey of pre-weaned calf housing and management was undertaken on dairy farms in Northern Ireland [11]. The objectives of this study are to highlight the key hygiene practices being undertaken for pre-weaned calves on these dairy farms and assess the impact of these practices on the level of bacteria within calf pens, feedstuffs and feeding equipment.

## 2. Materials and Methods

### 2.1. Farm Enrolment and Visits

Sixty-six dairy farms were visited as part of a survey on pre-weaned calf housing and management. Details pertaining to the selection and enrolment of farms are reported by Brown et al. [11]. Farms were visited on three pre-arranged occasions between 18 January 2019 and 2 May 2019. On the first visit, a face-to-face questionnaire was completed and measurements of housing were recorded. The questionnaire recorded information pertaining to nutrition, healthcare, housing practice and morbidity/mortality of pre-weaned calves. The second visit was arranged for the earliest suitable date after the first and the third visit took place two weeks after the second.

### 2.2. Questionnaire of Farm Management Practices and Housing Design

Details about the hygiene management of calf pens and feeding equipment were obtained from the farm manager or main calf rearer through a questionnaire [11]. Participants were asked if, and how often soiled bedding was removed from calf pens, how often pens were cleaned out entirely and the method used when cleaning. Where applicable, the type and amount of disinfectant and desiccant used when cleaning out pens was also recorded. Each pen in the calfhouses was assigned a score to indicate ease of cleaning using a standardized scoring system: (1) smooth surfaces, generally without tight gaps between frames and fittings and looks clean; (2) surfaces can be cleaned thoroughly but where 10% of surface areas are pitted or corroded; (3) surfaces show signs of cleaning but more than 10% has engrained dirt, and there is evidence of areas that are difficult to clean; and (4) visual evidence of cleaning difficulty, thick dirt on surfaces at calf height that has built up. The average ease of cleaning score was calculated for each farm to give one value per farm.

All pens in the calfhouse (range 1 to 40 across all farms) were assigned a drainage score using a standardized scoring system: (1) well-draining and mostly devoid of small cracks in the surface; (2) mostly well-draining with some cracking in floors and at joints to wall; (3) evidence of poor drainage ability, and cracking in surfaces; and (4) significantly poor drainage with broken surfaces and porous in parts. The main bedding type used in calf pens was recorded and the bedded proportion of each pen was broadly classified as either 100%, 75%, 50% or <50%. The type of floor material was specified as mesh, slats or concrete and where other materials were used, these were recorded. Concrete floors were further categorized as solid, with other floor types such as mesh, slats or woodchip classified as permeable. Where raised single pens with slats were placed on solid floors, these were classified as permeable. The internal area of pens and the number of calves housed in each pen were recorded to allow the calculation of pen space allowance (SA) (m^2^/calf). Average space allowance was calculated separately for single pens and group pens in each farm.

The presence of specific washing and drying areas for feeding equipment was recorded, as well as the type of feeding equipment used. Producers were asked how often feeding equipment was cleaned and what method was used to do this. The types and concentration of detergent used were not recorded in this study. Where automatic milk feeders (AMF) were used, the frequency and method of cleaning teats were documented.

The source of water used for feeding calves was recorded as the farm’s own source/bore well or from a mains supply. The ease of cleaning water drinkers/buckets from all occupied pens, up to a maximum of four pens, was assigned a score of 1 to 4. The scores were (1) drinker was located at the front of the pen, with a drain or removable, (2) drinker was located at the front of the pen, but without a drain or not removable, (3) drinker was located at the back of the pen and visually clean, and (4) drinker was located at the back of the pen and visually dirty. The average of these scores was then calculated to give a single score per farm.

### 2.3. Environmental Sampling

Swab samples of the calf environment, along with feed and water samples for analysis of hygiene parameters were collected on the second and third visits, which were approximately two weeks apart. A total of four samples of each type of feed (milk and concentrate) of water and of bedding were collected from each farm, two on each of the two visits. Up to eight swab samples of feeding equipment were collected on each farm, four on each visit. Water samples were collected from water troughs or buckets present in calf pens. A sterile 300 mL container was submerged into the trough and the sample then poured into a 500 mL container containing 20 g/mL Sodium Thiosulphate (DeltaLab Thiosulphate bottle 500 mL, DeltaLab, Barcelona, Spain). The type of pen (single or group), the location of the drinker (inside or outside the pen) and the type of drinker (bucket or self-fill drinker) were recorded. The time since the pen was last cleaned out or bedded was not recorded.

Liquid feed samples consisted of either milk replacer or whole milk samples. When the farm used milk replacer (MR), the farm manager or main calf-rearer prepared a sample using their routine method and two 300 mL subsamples were poured into sterile containers. If calves were fed using an automatic feeder (AMF), two 300 mL samples were collected from the AMF mixing container. If the farm used whole milk, two 300 mL samples of cows’ milk were collected directly from the bulk tank. Concentrate feed (CF) samples were collected from troughs present in calf pens. A sterile container was used to ‘scoop’ a sample from the tough or storage and the sample was then poured into a sealable, sterile bag. The source of CF was recorded as being either a bucket in a single pen or a trough in a group pen.

Swab samples were taken from the lower interior surfaces of feeders and buckets and the exterior of artificial teats. The swabbing technique consisted of vertical and horizontal strokes across a 25 cm^2^ area while gently rotating the swab (NRSII^TM^ Transwab^®^ 10 mL fill and swab, Medical Wire & Equipment Co Ltd., Corsham, UK). Upon collection of the sample, the swabs were returned to the 10 mL diluent container. As in the case of pen cleaning, the time since the equipment was last cleaned was not ascertained.

A sample of bedding was collected by first putting on a disposable plastic boot cover and then a sterile cotton net over the boot cover which was then walked across the pen floor in a ‘W’ shape with consistent paces. A 25 cm^2^ area of the centre heel of the sterile cotton net was then swabbed using horizontal and vertical strokes while rotating the swab. The type of pen (single or group) from which the bedding boot swab sample was taken from was recorded.

### 2.4. Microbiological Tests

Water samples were analysed within 24 h of collection and were stored at 4 °C from the point of collection to minimise the growth of micro-organisms before analysis. Samples were analysed by the Food Microbiology Unit in the Agri-Food and Biosciences Institute, (Belfast, Northern Ireland) for aerobic total viable count (TVC) as a measure of overall hygiene, and total coliform count (TCC) and *Escherichia coli* as measures of faecal contamination. Each sample was diluted 10^−1^ by mixing 10 mL of sample with 90 mL of maximum recovery diluent. The determination of TVC was completed by incubation of each sample on yeast extract agar, as a non-selective medium, at 22 °C (68 ± 4 h) and 37 °C (44 ± 4 h). The samples were incubated on membrane lauryl sulphate broth (MLSB) for determination of coliforms and *E. coli*. Then, 100 mL ± 5 mL of sample or diluted sample if needed, was filtered through a 0.45 µm pore size membrane onto petri dishes and was incubated at 30 °C for 4 ± 0.25 h, followed by further incubation at 37 °C or 44 °C for 14 ± 2 h to allow differentiation between coliforms and *E. coli*. For confirmation of coliforms and *E. coli*, colonies were sub-cultured into lactose peptone water (LPW) and incubated for 24 ± 1 h at the isolation temperature. These were further sub-cultured and incubated at 37 °C for up to 24 h onto MacConkey agar (MA) to determine purity and colony morphology and nutrient agar (NA) to select for lactose-fermenting Gram-negative bacteria. Confirmation of coliforms was by the oxidase test and of *E. coli* by the indole test. Colonies were counted and expressed as log_10_ colony forming units (CFU) per mL for TVC and as log_10_ CFU per 100 mL for TCC and *E. coli*.

MR, whole milk, CF and swab samples of bedding and feed equipment were cultured for TVC, TCC and *E. coli* using a spread plate method. The samples from the first 5 farms surveyed were analysed using a different method and are, therefore, not included in the final dataset.

For MR or whole milk samples, 0.7 mL of the sample was mixed with 0.3 mL of glycerol solution in a 1.5 mL micro-tube (Sarstedt AG & Co. KG, Nümbrecht, Germany). The CF samples were mixed with 0.3 mL glycerol. The swab containers were vortexed at 3000 rpm for 30 s to ensure the diluent mixed with the swab before extracting 0.7 mL of diluent and mixing with 0.3 mL of glycerol solution. All sample micro-tubes were vortexed at 3000 rpm for 30 s and transferred to −80 °C storage after 24 h in −20 °C where they remained until time of analysis. All the samples were analysed within 2 years of collection. Nutrient agar (Merck KGaA, Darmstadt, Germany) was the medium used for TVC and Coliform ChromoSelect Agar (Merck KGaA, Darmstadt, Germany) was used for the detection and enumeration of coliforms and *E. coli*. The samples were incubated at 24 °C for 48 h and the bacteria were counted. The arithmetic mean of each sample was calculated and once dilution into glycerol was accounted for, counts were expressed as log_10_ CFU per mL.

### 2.5. Statistical Analysis

All farm management practices and housing design parameters were inputted and summarized on a farm or pen basis using Microsoft Excel (Microsoft Corporation, Redmond, WA, USA). The sample source was recorded at the time of sample collection.

The sample source factors for the milk/MR samples were type (milk or MR) and source (milk from the bulk tank, manually prepared MR or MR from AMF mixer). The sample source factors for concentrate samples were single pen bucket or group pen trough. For the water samples, the farm housing and management factors were the source of water, average drinker hygiene score of less than or equal to 2, and average drinker ease of cleaning score of less than or equal to 2. An average score of 2 or less would indicate the more optimal 50% of available scores. The sample source factors were location of drinker (inside or outside of pen), type of drinker (manual-refill bucket or self-fill drinker) and type of pen (single or group).

For milk feeders, the sample source was the type of feeder (single or group). Similarly, for teats, the sample source was the type of feeder. Common farm housing and management variables for all types of feeding equipment were the presence of a separated cleaning area and drying area (yes/no). Nominal variables (yes/no) used in the model for milk replacer mixing buckets and teats were the use of hot water and chemical at cleaning, the use of hot water at cleaning, the use of chemical at cleaning, cleaning after every feed, cleaning daily, cleaning after every feed or daily with hot water and cleaning daily with chemical. Whether or not one or more mixing utensil sample from that farm had high TVC or high TCC was also used as a variable for milk feeders and teats.

Variables used in the model for bedding samples were the type of pen (single/group), the floor type (solid/permeable), average drainage score less than or equal to 2 (yes/no), if pens were cleaned at least every 3 weeks (yes/no) or at least every 6 weeks (yes/no), the use of disinfectant (yes/no), if pens were washed and disinfected at cleaning (yes/no), if pens were washed only at cleaning (yes/no), if pens were washed and disinfected within 3 week intervals (yes/no), or within 6 week intervals (yes/no), if pen space allowance was greater than 2 m^2^/calf or 3 m^3^/calf (yes/no) and if the average ease of cleaning score was less than or equal to 2 (yes/no).

Continuous TVC, TCC and *E. coli* data were categorised as above or below thresholds used to define hygiene targets for calf feed, feeding equipment and bedding. TVC targets in milk, MR and water were <4.0 log_10_ CFU/mL. Previous research has indicated a target of 3.0 log_10_ CFU/cm^2^ after sufficient cleaning [8,24], which equates to 3.4 log_10_ CFU/mL in this study. Due to the observational design of this survey and the large variation in cleaning methods seen on the farms, a target of 4.0 log_10_ CFU/mL was used, with the TVC target for milk/MR used as a reference for a maximum limit for target feeder levels. TCC and *E. coli* targets of <1.1 log_10_ CFU/mL (the detection limit) were used for milk, MR, concentrate feed and feeding equipment. For water, TCC and *E. coli* targets were <1.0 log_10_ CFU/100 mL. Care should be taken when comparing the boot swab hygiene results with other studies due to the variety of collection methods used, such as collected bedding sample or dilution of the entire boot cover [8]. In the absence of targets for calf pen bedding collected by the method employed in the current study, targets used for bedding boot swabs were <6.3 log_10_ TVC CFU/mL and <5.7 log_10_ TCC or *E. coli* CFU/mL, which were derived from bedding samples, as described by McGuirk [4]. The feeding equipment samples were classified as milk buckets/feeders, teats or mixing utensils due to the differences in their use and contact with calves, and thus were analysed separately.

**Figure 1:**
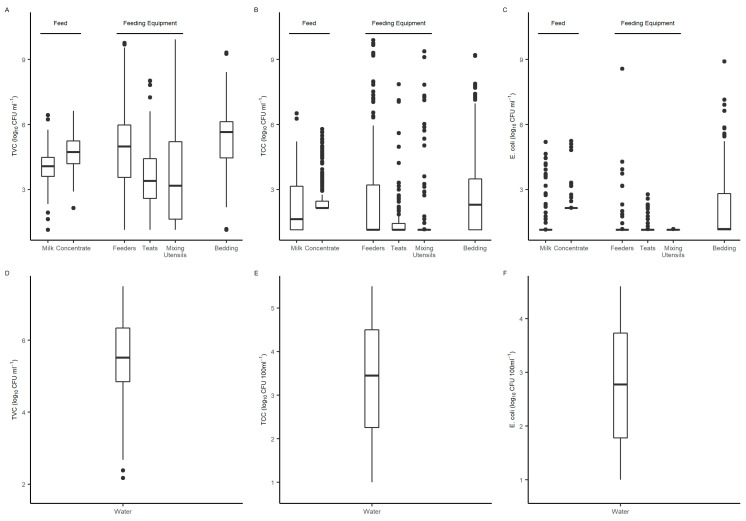
TVC (**A**,**D**), TCC (**B**,**E**) and E. coli (**C**,**F**) counts for calf milk/MR, concentrate feed, water, feeding equipment and bedding.

All the hygiene parameters with related farm management variables were entered into and analysed using Genstat^®^ (version 21, VSN International Ltd., Hemel Hempstead, UK). Boxplots were created to visually assess distributions (Figure 1). For each different sample type (milk, concentrate, water, feeder, teat, mixing equipment and bedding) each binary response variable was fitted against each variable (univariate) in turn using a generalized linear mixed model methodology with a binomial distribution, a logit link function and REML estimation. The sample source and farm housing and management factors were fitted as fixed effects in each case, and farm was included as a random effect in all models. The significance of the fixed effects was assessed by comparing a Wald statistic against the appropriate Chi-squared distribution.

Trends (*p* < 0.1), significant (*p* < 0.05) and highly significant associations (*p* < 0.01) of sample source or farm housing and management factors are displayed for feed (Table 3), feed equipment (Table 4) and bedding (Table 5). All other analyses are available in the Supplementary Material.

## 3. Results

### 3.1. Farm Management Characteristics Related to Hygiene

A greater overview of the dairy farms used in the survey is provided in Brown et al. [11]. Drinking water for calves was supplied from either a mains supply (47.0%, *n* = 31 farms) or from a bore well located on the farm (53.0%, *n* = 35 farms). The farm ease of cleaning score for water drinkers was on average 2.0 (range 1.0 to 4.0), whereas the farm hygiene score for water drinkers was 2.1 (range 1.0 to 3.3). Pre-weaned calves were offered milk replacer (81.8% of farms, *n* = 54) or whole milk (18.2% of farms, *n* = 12). None of the farms pasteurized whole milk prior to feeding calves.

A variety of methods was used to feed whole milk and MR with farms using more than one method: AMF (21.2%, *n* = 14), single teat feeders (51.5%, *n* = 34), group teat feeders (28.8%, *n* = 19), single buckets (37.9%, *n* = 25), and troughs (16.7%, *n* = 11). The most common frequency of manual (other than AMF) feed equipment cleaning was after every feed (38.1%, *n* = 24), and the use of hot water and chemical was the most common method for cleaning equipment, although the use of cold water (22.7%, *n* = 15) or hot water only (21.2%, *n* = 14) was also common (Table 1). A number of farms washed equipment with cold water after every feed (7.6%, *n* = 5) or on a daily basis (3.0%, *n* = 2) but washed with hot water and chemical on a weekly/fortnightly basis. The use of a washing area separated from livestock was observed on 34.9% (*n* = 23) of the farms, and a separated area for drying of cleaned equipment was used on 22.7% (*n* = 15) of the farms [11].

The number of calf pens varied on each farm from 1 to 55, with a mean of 15 pens. Of the 66 farms, 12 (18.2%) housed calves individually prior to weaning, 14 (21.2%) housed calves in groups from birth and the remaining 40 (60.6%) farms used single pens for a period of time but calves were grouped before weaning. The average number of calves in each group pen was 7 calves, ranging from 2 to 20 calves. The space allowance in single pens ranged from 0.9 to 3.2 m^2^ per calf with a mean of 1.5 m^2^ per calf. The mean space allowance in group pens was 3.4 m^2^ per calf, ranging from 1.3 to 8.7 m^2^ per calf. The variety of calfhouse floor materials found are described by Brown et al. [11]. The use of raised single pens with slats placed on solid concrete floors was commonplace (55.8% of the farms using single pens, *n* = 29). The farm average pen drainage score was 1.6 (95% CI: 0.8 to 2.5) and the ease of cleaning score for pens was an average of 1.9 (95% CI: 1.0 to 2.7).

Single-calf pens were most commonly cleaned every 3 weeks or less, although on 29% (*n* = 15) of farms using single pens, the interval between cleans was greater than 6 weeks (Table 2). Group pens were most commonly cleaned out at 3- to 6-week intervals. The most common method of cleaning was removal of organic matter, washing, and applying disinfectant, which was recorded on 38.5% (*n* = 20) of farms using single pens and 38.9% (*n* = 21) of farms using group pens. However, cleaning out and disinfecting only was a common practice for single pens (30.7%, *n* = 16), and 31.5% (*n* = 17) of farms using group pens did not wash or disinfect pens as part of the cleaning process. Disinfectant was used on 83.3% (*n* = 55) of the farms, and desiccants, namely lime-based products, were applied to calf pens on 45.5% (*n* = 30) of farms.

### 3.2. Key Characteristics of Hygiene

In total, 194 milk or MR samples, 223 concentrate samples, 216 water samples, 426 swab samples of feeding equipment and 230 boot swab samples were analysed for enumeration of hygiene indicators (TVC, TCC and *E. coli*). Within the 426 feeding equipment samples, 183 (42.9%) were from the inside of milk feeders or buckets, 110 (25.8%) were from the outside surface of teats and 133 (31.2%) were from feed preparation and mixing utensils (MU).

Coliforms were detected in the majority of water samples (94.0%), bedding samples (70.4%) and milk/MR samples (59.3%), and in 30.4% of concentrate samples and 32.8% of feeding equipment samples. *E. coli* was also detected in 89.8% of drinking water samples and, in 51.7% of bedding samples. *E. coli* was detected in a low percentage of milk/MR samples (14.9%), concentrate samples (5.8%) and feeding equipment (8.4%).

Coliforms were detected in approximately a quarter of MU samples (26.1%) and *E. coli* were detected in two MU samples (1.5%). Coliforms were detected in 45.4% and 29.1% of feeding buckets and teats, respectively. TVC, TCC and *E. coli* in milk feeders were all higher than those in teats (Figure 1). There were six samples from milk feeders with greater than 3.0 log_10_ CFU/mL *E. coli*. Of the 32 feeding equipment samples that had ≥6.0 log_10_ CFU/mL TCC, 26 came from 5 farms.

### 3.3. Factors Associated with Unsatisfactory Hygiene

As a large proportion (81.8%, *n* = 54) of the farms fed milk replacer to calves [11], and 80.4% (*n* = 156) of the collected liquid feed samples were of milk replacer (Table 3). Samples of whole milk tended to have a higher probability of being within the target range of TVC (4.0 log_10_ CFU/mL) (*p* < 0.1) compared with milk replacer samples. Samples of CMR from AMF tended to have increased probability of meeting TCC targets (<1.1 log_10_ CFU/mL) than samples of manually prepared CMR or milk samples (*p* < 0.1). The majority of concentrate samples (75.9%) had TVC levels above the target (4.0 log_10_ CFU/mL), but low proportions had coliforms and *E. coli* detected. Although no difference between pen types was seen in the TVC or TCC of concentrate samples, *E. coli* was more likely to be detected in samples from single pens (*p* < 0.05).

Considering the water samples, TVC were commonly (91.2%) above the target of 4.0 log_10_ CFU/mL. This trend was also observed for TCC and *E. coli* counts (target levels > 1.0 log_10_ CFU/100 mL. Water samples collected from buckets had a significantly higher probability (*p* < 0.05) of meeting target TVC levels than those collected from mounted, self-fill drinkers. A similar trend (*p* < 0.1) was observed for the TCC levels. Furthermore, the samples collected from group pens had a significantly lower probability of meeting target TCC levels (*p* < 0.05), although this is likely linked to the aforementioned trend as 100% of self-fill drinkers were found in group pens. Average cleaning ease scores of buckets/drinkers of less than 2, tended (*p* < 0.1) to relate to a higher probability of water samples having target TVC levels. Source of water (main/bore well) and situation of the drinker (inside/outside pen) had no effect on the probability of samples having target TVC, TCC or *E. coli* levels.

The presence of a separate cleaning or drying area for milk/MR mixing and feeding utensils had no impact on the probability of TVC, TCC or *E. coli* being within the target range for any feeding equipment types. The type of feeder (single calf or group feeder) was not related to the probability of TVC, TCC or *E. coli* being within the target for milk feeders or teats. Milk feeders that were cleaned after every feed had a significantly higher probability (*p* < 0.05) of being within the target TVC range (Table 4). Washing with chemical on a daily basis had a tendency to increase the likelihood of being within the target TCC in milk feeders. However, the use of hot water or chemical within washes, regardless of frequency, was not related to the probability of milk feeders being within the target TVC or *E. coli*. Where at least one swab sample of mixing utensils had TCC detected, there was a lower probability of target TVC (*p* < 0.05) or TCC (*p* < 0.05) in milk feeders.

No relationship was observed between the frequency of cleaning teats and the probability of target hygiene indicator levels. On the other hand, cleaning with hot water and chemical was associated with a lower likelihood of coliform detection (*p* < 0.05).

Within the bedding samples collected, the majority were within target ranges for TVC (77.4%), TCC (90.4%) and *E. coli* (97.0%). Samples from group pens were significantly more likely (Table 5; *p* < 0.01) to be within the TVC target (6.3 log_10_ CFU/mL) than those from single pens. This was also the case with TCC (Table 5; *p* < 0.01). Neither the frequency nor the method of cleaning was related to the probability of target TVC, TCC or *E. coli*. Floor type was highly associated (Table 5; *p* < 0.01) with the probability of being within the target range for TCC, where samples from pens with permeable floors were less likely to be within the target. A trend (Table 5; *p* < 0.1) was seen in the relationship between optimal drainage scores (avg. ≤ 2) and increased likelihood of being within the target TCC range. A space allowance of ≥2 m^2^/calf was significantly associated with increased probability of target TCC, but not TVC or *E. coli*. None of the factors measured were related to the probability of being within the target range for *E. coli (*Table 5*)*.

## 4. Discussion

Young calves are extremely susceptible to disease as they are still developing immunocompetence and because of the numerous stressors that they face in their first few weeks of life (e.g., dam separation, disbudding, commingling) [2]. Therefore, minimizing their exposure to disease-causing organisms through appropriate hygiene is an essential part of pre-weaned calf management [8].

In this study, samples were collected from potential sources of pathogenic bacteria to assess micro-organism counts. As the range of TVC, TCC and *E. coli* counts were large (Figure 1) and a proportion of samples had counts much higher than levels suitable for young calves, categorization of counts allowed a more relevant evaluation to be made. A risk-factor approach was used to evaluate whether relevant farm management factors impacted the likelihood of hygiene indictor counts being within appropriate target levels.

### 4.1. Liquid and Solid Feed Hygiene

Poor hygiene of calf liquid feed may increase exposure to enteric disease-causing pathogens. Furthermore, high levels of microbial contamination may also impact nutrient absorption [25]. Bacterial contamination of colostrum is thought to disrupt nutrient uptake, either by binding of free proteins in the gut lumen or by blocking receptors for transport across the intestinal epithelium [25]. The reduction in nutrient utilisation and changes in metabolic pathways are also caused by immune stimulation when animals are exposed to environmental antigens [26]. This was exemplified by reductions in the growth of pigs reared in unhygienic conditions compared with those reared in clean conditions [27].

Although acceptable targets of 5.0 log_10_ CFU/mL for TVC and 4.0 log_10_ CFU/mL for TCC have been used for calf milk hygiene in previous studies [28,29], the 5.0 log_10_ CFU/mL target was first described in relation to colostrum [30], for which the harvesting/preparation conditions allow for greater contamination of feeds, when compared with milk replacer or cow’s milk collected in the milking parlour. The value of 4.0 log_10_ CFU/mL for TVC [4] was subsequently used. In 52.1% of milk/MR samples within this study, TVC were above 4.0 log_10_ CFU/mL. However, only 2.6% were above 5.0 log_10_ CFU/mL, so approximately 50% of these samples had TVC between 4.0 and 5.0 log_10_ CFU/mL.

In the current study, MR samples collected from AMF mixing jars tended to have a higher probability of being within target TCC range than milk samples and manually prepared MR samples (Table 3). The risk of coliforms is higher in milk samples than in MR samples, due to potential variations in hygiene practices of milk collection and hygiene in the milking plant [31]. In relation to the differences between AMF-prepared and manually prepared MR samples, it must be considered that many current AMF models include cleaning functions [29], which may be a factor in the lower probability of coliform detection. The contamination of manually prepared MR can take place either in storage, during preparation or potentially through the water that was mixed with the milk powder. As water samples collected in this study were from drinkers in calf pens, meaning contamination by calves was likely, these samples cannot be considered with regard to water used to prepare MR. Storage of unsealed MR powder and its impact on hygiene remains largely unstudied. As herd size (66 to 400 cows) and total number of pre-weaned calves at a given time varied within this study [11], the rate at which it is used during the calving season may influence the risk of exposure leading to bacterial contamination of stored MR.

The hygiene of calf starter feed is unstudied despite the clear importance of starter feed intake for successful development of the rumen [32,33]. The acceptable TVC target was set at 4.0 log_10_ CFU/mL (calculated to be equivalent to CFU/g in this study) due to the lack of clear solid feed targets and as it was comparable to the TVC targets used for milk/MR and water. A lower probability of detecting *E. coli* in the group pen trough starter feed than in single pen buckets is of interest. Single pens generally accommodate younger calves as grouping typically occurs as calves’ age. This finding may be due to lower intakes of starter feed and potentially longer durations between fresh offerings of starter feed during which time it could become contaminated. Additionally, smaller space allowances in single pens in this study may lead to increased risk of defecation on side walls and in feed buckets. However, as only 5.8% of concentrate feed samples had *E. coli* present, it was not a common occurrence, and therefore, may not be of great consequence.

In previous research, the presence of particular strains of *E. coli* (O157) has been observed in samples from stored concentrate, prior to being offered to cattle [34]. One potential source of contamination suggested was the mixed use of equipment and areas for handling of feed and livestock manures. Cross-contamination of equipment was possible on any of the farms in the current study; however, evaluation of this is difficult.

The presence of *E. coli* in starter feed is indicative of faecal contamination and is relevant to calf gut health as more common diarrhea-causing pathogens such as *C. parvum* may also be transmitted via this route [35]. When colostrum intake has been limited, as was likely in 22.8% of farms in the current study that fed less than 3 L in the first feed, there may be a lack of intestinal microbiota diversity, which increases susceptibility to colonization by pathogenic bacteria [36]. Furthermore, faecal contamination of concentrate feed is likely to have an effect on palatability and voluntary intakes, similar to that observed in water [22,37]. These findings add weight to the advice to provide small portions of fresh feed frequently to calves during the first weeks of life [38].

Water consumption encourages intake of solid feed [23] as a means of supporting early rumen development prior to weaning [39]. On approximately 68% of the farms in this study, water was offered to calves at birth, with the first offering occurring at an average of 4 days of age across all farms. Further to its role in promoting solid feed intake, provision of water to young calves influences the early colonization of microbiota in the rumen [40]. TVC, TCC and *E. coli* counts in water samples were highly variable and the median values were much higher than advisory levels quoted in the scientific literature or by industry [38,41]. This indicates that the hygiene standards of water provided to pre-weaned calves within this study pose a risk to animal health. Water is a key vector of disease for livestock, as well as humans, and poor quality is linked with diminished cattle performance [22]. Water troughs, and water sources in general, are shown to be reservoirs of *E. coli* O157:H7, *Cryptosporidium* and Coccidia [42,43,44]. As hygiene management of calves regularly involves the cleaning and disinfection of pens and feeding equipment [8], such practices are also required for drinking water sources.

In the current study, water from self-fill drinkers had a significantly lower probability of being within target TVC levels than water from manually filled buckets. A similar trend was observed in water TCC. As buckets that are manually filled require regular observation by the calf-rearer, it is possible that cleanliness is also observed and thus maintained. Conversely, self-fill drinkers are less likely to be routinely checked for function and subsequent hygiene. Additionally, self-fill drinkers were mounted within calf pens and could not be easily moved elsewhere for cleaning and disinfection. They are, in comparison, more complex designs than buckets, so ease and effectiveness of cleaning is reduced. This is reinforced in that a trend was observed between average ease of cleaning scores of ≤2 and increased probability of target TVC. Increasing the ease of cleaning is a key factor in the motivation to complete cleaning and disinfection routinely [45]. Furthermore, motivation to clean drinkers may be influenced by how the producer perceives the benefit of cleaning drinkers in terms of disease risk [45]. Water quality is a relatively unstudied factor in calf health, and disease and quality guidelines are fairly non-specific [46]; thus, its role as a vehicle for disease transmission may be under-appreciated by producers.

The probability of being within the target drinking water TCC range was significantly higher in single pen drinkers than in group pen drinkers, possibly due to the increased number of animals accessing the same drinking sources in group pens. However, the fact that self-fill drinkers were only located in group pens means that drinker type may have influenced these findings. No significant difference in target water quality was seen between the sources of water for drinkers, indicating that contamination by calves in the pen is a more important factor. This was observed in another study assessing the microbiological quality of water from adult cattle drinkers [46]. In the current study, low average hygiene scores were not associated with the probability of any target hygiene indicator levels. This may be due to differences in quality over time, as observations were made on the first visit, and samples collected on visits 2 and 3.

These findings highlight the risk of disease transmission to young calves by drinking water, and the high level of exposure to faecal contamination within drinkers. Drinker hygiene should be a part of routine cleaning and disinfection and the quality of water monitored as a risk factor in cases of prevalent enteric disease in calf groups.

### 4.2. Feeding Equipment Hygiene

Within this study, a large variation in the methods and frequency of cleaning milk feeding equipment was observed. As it is important that milk/MR contains low TVC and minimal levels of coliforms and *E. coli*, the same is required for the equipment used to prepare and feed it. TVC were greater and coliforms were more commonly detected inside milk feeders than on the outside surface of teats.

Milk feeders/buckets had a significantly higher probability of being within the target TVC range (<4.0 log_10_ CFU/mL) when equipment was washed after every feed, as would be expected. This finding highlights the importance of cleaning equipment after every feed to minimise the accumulation of micro-organisms and biofilms on feeding equipment. However, this result conflicts with previous research [8] that showed the opposite effect on milk feeder TVC; however, in that study, disassembling of equipment was associated with increased odds of being within the target for TVC, and various swab locations were included within the ‘feeding equipment’ category, including inside/outside of teats. For the teats sampled in this study, cleaning after every feed was not related to the probability of TVC being within targets, similar to the previous finding, suggesting that more intense practices involving disassembly of teats are required to reduce TVC in teats. The proportion of farms cleaning feeders/buckets after every feed (38.4%) is much lower than that reported in a study of Austrian dairy farms (97%) [47] but is similar to a study of German farms (36%) [8]. As the majority of farms did not wash equipment after each feed, it is clear that large reductions in TVC could be achieved by advocating the need to clean after each use.

Daily washing with chemical led to a higher probability of being within the target TCC range. Similarly, washing with hot water and chemical tended to increase the probability of target range TCC in teats. This is supported by the findings of Hyde et al. [48], who reported that the use of various chemicals significantly reduced (peracetic acid), or tended to reduce (hypochlorite or soap) total bacterial count (TBC; a measure of all cells that are both alive and dead), when compared with water only. Using peracetic acid also significantly reduced TCC, whereas hypochlorite tended to reduce TCC [48]. Similarly, washing with hot water significantly reduced TBC [48]. These findings highlight the requirement for chemical inclusion in routine hygiene management due to its ability to breakdown biofilms and remove pathogens [9]. A limitation of the current study was that the type and concentration of detergents used for cleaning feeding equipment were not recorded. Such information may have highlighted variability or trends in common practice relating to detergent concentration and may have helped explain variation in hygiene indicator detection.

Where at least one sample of feed preparation equipment had a high TCC, there was a significant probability that milk feeders or buckets would have TVC or TCC outside of the target range. This relationship may not be causative and may represent a general indication of suboptimal hygiene on farms. The few associations between cleaning practice and target hygiene indicators may be due to common dirtying of post-cleaned feeders. Cross-contamination of feeders is likely considering that over 65% of farms cleaned equipment in areas where livestock were present, and 77% left equipment to dry in these areas. Splashing of contaminated water was suggested as a possible route of re-contamination of pig feeders in previous studies [49,50]. Although it was not possible to evaluate in the current study, Barry et al. [3] found that hygiene scores of feeding equipment tended to be lower at the end of the calving season than at the beginning, suggesting that the methods employed were not sufficient. This may be related to the fact that none of the farms in that study washed equipment after every feed, which as observed is a key factor in maintaining lower TVC [8].

### 4.3. Bedding Hygiene

As previously suggested by Heinemann et al. [8], the impact of varied methodologies for collection of bedding swab samples must be considered, and therefore, results should be compared with caution. Within this study, a general observation was that increased space per calf was linked with a higher likelihood of hygiene indicators being within the target range. The increased probability of target level TVC and TCC in group pens (compared with single-calf pens) was likely related to that of SA ≥2 m^2^/calf increasing the probability of target TCC. The space allowance in single-calf pens (median 1.3 m^2^/calf) was lower than that in group pens (median 3.2 m^2^/calf). Although no research has previously evaluated the impact of space allowance/stocking density in the context of hygiene for dairy calves, relationships have been observed between lower stocking densities and reduced adverse health events [15] and reduced probability of abnormal eye and ear scores [29]. Similarly, within studies including older cattle, reducing stocking density has been seen to increase the live weight gain (LWG), slaughter weights and feed conversion efficiency of fattened beef cattle [51], and improve feed intake, LWG and improve hoof health of dairy heifers [52]. The current study did not evaluate the impact of stocking density or microbial loads on the health or mortality of dairy calves. Including these variables would have allowed a more in-depth analysis of the implications of these factors for calf health. A consensus throughout housing of all livestock species is that increasing SD reduces air quality [14,53,54]. It is perceived to be a more important determinant of air quality in calf housing than ventilation rate [55], as accounting for doubling the stocking density would require approximately ten times the rate of ventilation [14,53]. As the two primary sources of airborne bacteria are the animals and the pen, a relationship between bedding and air quality may be observed in that bacteria from bedding may be aerosolized when disturbed [14].

Although not evaluated in the current study, the frequency of adding fresh bedding is of importance to calf pen bedding hygiene. Increased frequency of adding bedding to automatic feeder pens was associated with a decrease in the prevalence of calf diarrhea [28]. However, the risk of disturbing settled pneumonia pathogens within bedding is not mitigated by addition of bedding alone, as an increased depth of wet-packed bedding in pens was associated with the prevalence of BRD [28]. Therefore, the regular removal of contaminated, wet bedding, as well as addition of fresh, dry bedding is a major factor in maintaining a clean environment for rearing calves [56].

A further observation was that solid-floored pens had increased probability of being within the target TCC range when compared with pens with permeable floors. This finding may be contrary to the concept that improved drainage reduces floor and bedding moisture and subsequently reduces the viability of microbes. However, as a large proportion of permeable floor pens were raised, slatted, single pens, these factors may be confounded, in that the predominant factor was the space allowance/calf of the pen, rather than its ability to drain.

As a very low proportion (3%) of the samples had *E. coli* above the target level (5.7 log_10_ CFU/mL), no relationships between farm management factors and probability of bedding within the *E. coli* target were observed. The large variation in frequency and methods of cleaning calf pens may indicate a lack of clear consensus with regard to the optimum parameters of cleaning among calf-rearers, or perhaps results from a variety of time constraints or calf accommodation availability [57]. As the pens sampled in this study were occupied, the method and frequency of cleaning calf pens was not seen to influence the probability of TVC, TCC or *E. coli* being within the target. Sample collection from pen surfaces taken immediately after cleaning and disinfection would yield more valuable information such as the efficacy of various cleaning protocols [8].

## 5. Conclusions

Within the farms in this study, much variation was observed in the practices of cleaning milk feeding equipment and pens. This highlights that there may be a lack of clear consensus as to the most effective practices amongst NI dairy farmers or that there is variation in the availability of time and labour to complete hygiene related tasks. Bacterial counts in water samples were largely outside of target ranges and may indicate a common vector in the faecal–oral transmission of calf enteric pathogens. Water hygiene was poorer in self-fill drinkers and tended to be poorer where the ease of cleaning was reduced; thus, promoting drinker cleaning routines may aid in reducing bacterial counts. Milk replacer samples from automatic feeders tended to have lower TCC than whole milk or manually prepared milk replacer samples, suggesting automatic sanitation of feeders may provide a hygiene benefit. Faecal contamination of starter feed was more likely in single-calf pens than in groups, which highlights a need to ensure fresh feed is offered to young calves frequently.

The TVC of milk feeders were only observed to be reduced by cleaning after every feed, whereas no difference in probability of target bacterial counts were seen with use of chemical or less frequent cleans (daily). The TCC of teats was positively related to washing with hot water and detergent. When coliforms were detected on mixing utensils, TVC and TCC of milk feeders were more likely to be greater than the target ranges. Bacterial counts of bedding samples in this study were not impacted by cleaning of pens or the use of disinfectant. However, TCC was increased by reduced space allowance in pens and solid floors under bedding. Single-calf pens had higher TVC and TCC than group pens, which may be related to space allowance. Thus, ensuring calves are provided with adequate pen space may be of benefit to health. The results from this study demonstrate that there are numerous sources of pathogenic exposure to young calves and that many of these sources could be mitigated through use of standardized protocols.

## Figures and Tables

**Table 1:** Distribution of 66 farms according to the method and frequency of cleaning milk feeding equipment and type of milk feed.

Feeding Equipment Cleaning Method	Every Feed	Daily	Every 1–2 Weeks	≥Monthly	Unknown	Total ^1^
Hot water + chemical	3	0	7	8	0	**18**
Hot water only	8	2	3	1	0	**14**
Cold water + infrequent hot water + chemical	5	2	0	0	0	**7**
Cold water + chemical	1	3	2	2	1	**9**
Cold water only	7	1	4	2	1	**15**
Not cleaned	0	0	0	1	0	**1**
Unknown	0	0	0	1	1	**2**
**Total**	**24**	**8**	**16**	**15**	**3**	**66**

^1^ Descriptive summary of all 66 farms, including the 5 farms that were excluded from the model.

**Table 2:** Distribution of 66 farms according to the method and frequency of cleaning single and group calf pens.

Calf Pen Cleaning Method	≤3 Weeks		3 < x ≤ 6 Weeks		>6 Weeks		Unknown		Total ^3^	
	S ^1^	G ^2^	S	G	S	G	S	G	S	G
Cleaned out only	4	9	1	6	2	0	2	2	**9**	**17**
Cleaned out and washed	5	0	1	2	1	0	0	1	**7**	**3**
Cleaned out and disinfectant/desiccant	9	1	2	4	5	5	0	1	**16**	**11**
Cleaned out, washed and disinfectant/desiccant	6	3	7	8	7	8	0	2	**20**	**21**
Unknown	0	0	0	0	0	0	0	2	**0**	**2**
**Total**	**24**	**13**	**11**	**20**	**15**	**13**	**2**	**8**	52 *	54 **

^1^ Single pen, ^2^ Group pen. ^3^ Descriptive summary of all 66 farms, including the 5 farms that were excluded from the model. * 52 of 66 farms used single pens for pre-weaned calves. ** 54 of 66 farms used group pens for pre-weaned calves.

**Table 3:** Sample source and farm housing and management factors for liquid and solid feed offered to calves that impacted the likelihood of meeting hygiene indicator targets.

Variable (Hygiene Indicator)	% Within Target	Total Number	Probability * (LCI-UCI **)	*p*-Value
**Milk/Milk replacer**				
Type (TVC ^1^)				
Cow’s milk	64.9	37	0.61 (0.37–0.81)	0.06
Milk replacer	35.9	137	0.35 (0.24–0.48)	
Source (TCC ^1^)				
Bulk tank	23.7	38	0.23 (0.10–0.45)	0.05
AMF prepared	64.5	31	0.63 (0.38–0.83)	
Manually prepared	35.5	124	0.34 (0.24–0.47)	
**Starter feed**				
Pen source (*E. coli* ^1^)				
Single bucket	89.4	85	0.89 (0.77–0.95)	0.03
Group trough	97.1	138	0.97 (0.92–0.99)	
**Water**				
Drinker Type (TVC ^1^)				
Self-fill drinker	2.7	110	0.03 (0.01–0.09)	0.02
Bucket	13.5	104	0.14 (0.07–0.26)	
Farm avg. cleanability Score ≤ 2 (TVC ^1^)				
Yes	8.8	147	0.11 (0.06–0.19)	0.07
No	1.6	63	0.02 (0.00–0.11)	
Pen type (TCC ^2^)				
Single	9.7	62	0.12 (0.05–0.27)	0.03
Group	3.3	152	0.03 (0.01–0.08)	
Drinker Type (TCC ^2^)				
Self-fill drinker	1.8	110	0.02 (0.01–0.09)	0.07
Bucket	8.7	104	0.09 (0.04–0.20)	

^1^ log_10_ CFU/mL, ^2^ log_10_ CFU/100 mL. * Probability of meeting pre-specified hygiene targets. ** Lower and upper 95% confidence intervals.

**Table 4:** Sample source and farm housing and management factors for milk feeding equipment that impacted likelihood of meeting hygiene indicator targets.

Variable (Hygiene Indicator)	% Within Target	Total Number	Probability * (LCI-UCI **)	*p*-Value
**Milk Feeders**				
Cleaned after every feed (TVC ^1^)				
Yes	49.1	55	0.47 (0.29–0.66)	0.04
No	24.1	116	0.23 (0.14–0.36)	
≥1 Mixing utensil item TCC ^2^ High (TVC ^1^)				
Yes	43.0	49	0.17 (0.07–0.35)	0.03
No	14.3	93	0.43 (0.29–0.59)	
Washed with Chemical daily (TCC ^1^)				
Yes	77.8	18	0.79 (0.50–0.94)	0.07
No	50.7	150	0.51 (0.41–0.61)	
≥1 Mixing utensil item TCC High (TCC ^1^)				
Yes	38.8	49	0.38 (0.22–0.56)	0.05
No	61.1	95	0.62 (0.48–0.73)	
**Teats**				
Washed with hot water and chemical (TCC ^1^)				
Yes	88.2	34	0.87 (0.68–0.96)	0.07
No	64.5	76	0.66 (0.51–0.78)	

^1^ log_10_ CFU/mL. ^2^ At least one sample of mixing utensil had coliforms detected. * Probability of meeting pre-specified hygiene targets. ** Lower and upper 95% confidence intervals.

**Table 5:** Sample source and farm housing and management factors for pen bedding that impacted likelihood of meeting hygiene indicator targets.

Variable (Hygiene Indicator)	% Within Target	Total Number	Probability * (LCI-UCI **)	*p*-Value
Pen type (TVC ^1^)				
Single	64.8	88	0.66 (0.52–0.78)	0.01
Group	84.1	132	0.84 (0.74–0.90)	
Pen type (TCC ^1^)				
Single	88.1	93	0.80 (0.64–0.91)	<0.01
Group	93.5	135	0.96 (0.89–0.98)	
Floor Type (TCC ^1^)				
Solid	95.3	128	0.96 (0.88–0.99)	<0.01
Permeable	84.0	100	0.76 (0.52–0.91)	
Drain score ≤ 2 (TCC ^1^) ^2^				
Yes	93.6	173	0.93 (0.86–0.97)	0.09
No	80.0	55	0.79 (0.52–0.93)	
SA > 2 m^2^/calf (TCC ^1^) ^3^				
Yes	93.5	108	0.94 (0.84–0.98)	0.04
No	80.0	91	0.79 (0.60–0.91)	

^1^ log_10_ CFU/mL. ^2^ Average drainage score of all pens in calfhouse. ^3^ Pen space allowance for calves. * Probability of meeting pre-specified hygiene targets. ** Lower and upper 95% confidence intervals.

## Data Availability

The data presented in this study is available on request from the corresponding author.

## Supplementary Materials

The following supporting information can be downloaded at: https://www.mdpi.com/article/10.3390/ani13061109/s1, Table S1: Risk factors for liquid and solid feed offered to calves that were not associated with the likelihood of meeting hygiene indicator targets; Table S2. Risk factors for bedding that were not associated with the likelihood of meeting hygiene indicator targets; Table S3. Risk factors for feeding equipment that were not associated with the likelihood of meeting hygiene indicator targets.
