# Supplementary material for: Pre-Weaned Calf Rearing on Northern Irish Dairy Farms—Part 2: The Impact of Hygiene Practice on Bacterial Levels in Dairy Calf Rearing Environments
Source: Animals (Basel). 2023 Mar 21;13(6):1109. doi: 10.3390/ani13061109 (PMC10044673; doi:10.3390/ani13061109)
Supplement: Supplementary file 1 [file animals-13-01109-s001.zip › animals-2069549-supplementary.pdf]

Supplementary Table S1. Risk factors for liquid and solid feed offered to calves that were not associated with the likelihood of meeting hygiene indicator targets

Variable (Hygiene Indicator)	% within target	Total number	Probability* (LCI-UCI**)	p-value
Milk/Milk replacer				
Source (TVC)				
Bulk tank	64.9	37	0.61 (0.37-0.81)	0.172
AMF prepared	40.7	27	0.37 (0.16-0.65)	
Manually prepared	34.5	110	0.34 (0.22-0.49)	
Type (TCC)				
Cow's milk	23.7	38	0.23 (0.10-0.45)	0.147
Milk replacer	81.9	155	0.40 (0.30-0.52)	
Source (*E. coli*)				
Bulk tank	63.2	38	0.64 (0.38-0.83)	0.300
AMF prepared	80.6	31	0.77 (0.50-0.92)	
Manually prepared	82.3	124	0.82 (0.71-0.90)	
Type (*E. coli*)				
Cow's milk	63.2	38	0.64 (0.39-0.83)	0.129
Milk replacer	77.8	155	0.81 (0.71-0.89)	
Starter feed				
Pen source (TVC)				
Single bucket	13.3	75	0.12 (0.05-0.26)	0.804
Group trough	11	118	0.11 (0.05-0.21)	
Pen source (TCC)				
Single bucket	62.4	85	0.62 (0.47-0.75)	0.127
Group trough	73.9	138	0.74 (0.63-0.83)	
Water				
Drinker source (TVC)				
Mains supply	10.7	103	0.11 (0.05-0.24)	0.411
Bore well	7.1	113	0.07 (0.03-0.16)	
Drinker location (TVC)				
Inside pen	8.1	149	0.08 (0.04-0.16)	0.982
Outside pen	7.7	65	0.08 (0.03-0.20)	
Pen type (TVC)				
Single calf pen	12.9	62	0.14 (0.06-0.30)	0.107
Group calf pen	5.9	152	0.06 (0.03-0.12)	
Average Hygiene score (TVC)				
≤2	7.1	140	0.10 (0.05-0.18)	0.313
>2	4.3	70	0.05 (0.01-0.15)	
Drinker Source (TCC)				
Mains supply	7.8	103	0.08 (0.03-0.18)	0.396
Bore well	4.4	113	0.04 (0.02-0.12)	
Drinker location (TCC)				
Inside pen	6.7	149	0.06 (0.03-0.13)	0.447
Outside pen	1.5	65	0.04 (0.01-0.14)	
Average Hygiene score (TCC)				
≤2	1.4	140	0.04 (0.01-0.09)	0.306
>2	7.1	70	0.07 (0.03-0.17)	
Average Clean-ease score (TCC)				
≤2	2.0	147	0.04 (0.02-0.09)	0.547
>2	6.3	63	0.06 (0.02-0.017)	

Drinker Source (*E. coli*)				
Mains supply	13.6	103	0.14 (0.06-0.27)	0.249
Bore well	7.1	113	0.07 (0.03-0.16)	
Drinker location (*E. coli*)				
Inside pen	11.4	149	0.10 (0.05-0.19)	0.661
Outside pen	4.6	65	0.08 (0.03-0.21)	
Pen type (*E. coli*)				
Single calf pen	11.3	62	0.14 (0.06-0.30)	0.274
Group calf pen	8.6	152	0.08 (0.04-0.16)	
Drinker type (*E. coli*)				
Self-fill drinker	7.3	110	0.07 (0.03-0.16)	0.289
Bucket	11.5	104	0.13 (0.06-0.25)	
Average Hygiene score (*E. coli*)				
≤2	4.3	140	0.06 (0.03-0.12)	0.105
>2	14.3	70	0.14 (0.07-0.27)	
Average Clean-ease score (*E. coli*)				
≤2	5.4	147	0.07 (0.03-0.14)	0.336
>2	12.7	63	0.12 (0.05-0.27)	

TVC = Total Viable Count TCC = Total Coliform Count

Supplementary Table S2. Risk factors for bedding that were not associated with the likelihood of meeting hygiene indicator targets

Variable (Hygiene Indicator)	% within target	Total number	Probability* (LCI-UCI**)	p-value
Disinfectant used (TVC)				
Yes	75.7	185	0.76 (0.66-0.84)	0.720
No	80.0	35	0.80 (0.55-0.93)	
Floor Type (TVC)				
Solid	78.9	123	0.80 (0.69-0.88)	0.292
Permeable	71.4	97	0.72 (0.58-0.83)	
Drain score (TVC)				
≤2	80.1	166	0.80 (0.70-0.87)	0.155
>2	68.5	54	0.66 (0.45-0.82)	
Clean <6 weeks (TVC)				
Yes	77.0	122	0.77 (0.65-0.85)	0.591
No	70.1	67	0.72 (0.55-0.84)	
Clean <3 weeks (TVC)				
Yes	77.4	53	0.78 (0.59-0.89)	0.660
No	73.5	136	0.74 (0.62-0.82)	
Wash and Disinfect <6 weeks (TVC)				
Yes	74.3	74	0.73 (0.56-0.85)	0.716
No	75.2	133	0.76 (0.64-0.85)	
Wash and Disinfect <3 weeks (TVC)				
Yes	76.0	25	0.77 (0.50-0.92)	0.859
No	74.7	182	0.75 (0.65-0.83)	
Wash <6 weeks (TVC)				
Yes	74.4	43	0.74 (0.52-0.88)	0.917
No	74.7	146	0.75 (0.64-0.83)	
Wash <3 weeks (TVC)				

Yes	84.7	13	0.83 (0.47-0.97)	0.538
No	73.9	176	0.74 (0.64-0.82)	
Wash and Disinfect (TVC)				
Yes	73.3	75	0.73 (0.57-0.85)	0.744
No	75.8	132	0.76 (0.64-0.85)	
SD >2 m^2 (TVC)				
Yes	73.3	105	0.74 (0.61-0.84)	0.497
No	80.5	87	0.79 (0.66-0.85)	
Cleanease score (TVC)				
≤2	74.7	162	0.75 (0.63-0.83)	0.468
>2	81.0	81	0.81 (0.63-0.92)	
Disinfectant used (TCC)				
Yes	90.6	191	0.90 (0.81-0.95)	0.708
No	89.2	37	0.87 (0.56-0.97)	
Clean <6 weeks (TCC)				
Yes	92.1	127	0.91 (0.81-0.96)	0.313
No	84.3	70	0.84 (0.64-0.94)	
Clean <3 weeks (TCC)				
Yes	89.5	57	0.88 (0.68-0.96)	0.963
No	89.3	140	0.89 (0.77-0.95)	
Wash and Disinfect <6 weeks (TCC)				
Yes	92.0	79	0.91 (0.76-0.97)	0.676
No	88.3	136	0.88 (0.86-0.95)	
Wash and Disinfect <3 weeks (TCC)				
Yes	93.1	29	0.91 (0.61-0.98)	0.862
No	89.2	186	0.89 (0.80-0.95)	
Wash <6 weeks (TCC)				
Yes	91.7	48	0.91 (0.70-0.98)	0.654
No	88.6	149	0.88 (0.76-0.94)	
Wash <3 weeks (TCC)				
Yes	100.0	17	1.00	0.978
No	88.3	180	0.88 (0.78-0.93)	
Wash and Disinfect (TCC)				
Yes	86.2	80	0.87 (0.69-0.95)	0.552
No	91.9	135	0.91 (0.80-0.96)	
Cleanease score (TCC)				
≤2	92.3	169	0.90 (0.79-0.95)	0.892
>2	84.7	59	0.91 (0.71-0.97)	
Pen type (*E. coli*)				
Single	97.8	93	0.96 (0.88-0.99)	0.682
Group	95.7	136	0.97 (0.92-0.99)	
Disinfectant used (*E. coli*)				
Yes	97.4	191	0.97 (0.93-0.99)	0.520
No	94.7	38	0.95 (0.73-0.99)	
Floor Type (*E. coli*)				
Solid	98.4	128	0.99 (0.93-1.00)	0.146
Permeable	95.0	101	0.94 (0.82-0.98)	
Drain score (*E. coli*)				
≤2	97.1	173	0.97 (0.92-0.99)	0.841

>2	96.4	56	0.96 (0.82-0.99)	
Clean <6 weeks (*E. coli*)				
Yes	97.6	127	0.98 (0.91-0.99)	0.606
No	95.7	70	0.96 (0.84-0.99)	
Clean <3 weeks (*E. coli*)				
Yes	94.8	57	0.95 (0.79-0.99)	0.402
No	97.9	140	0.98 (0.92-0.99)	
Wash and Disinfect <6 weeks (*E. coli*)				
Yes	100	79	1.00	0.987
No	94.9	127	0.95 (0.88-0.98)	
Wash and Disinfect <3 weeks (*E. coli*)				
Yes	100	79	1.00	0.991
No	94.9	137	0.96 (0.91-0.99)	
Wash <6 weeks (*E. coli*)				
Yes	100	48	1.00	0.997
No	96	149	0.96 (0.90-0.99)	
Wash <3 weeks (*E. coli*)				
Yes	100	48	1.00	0.997
No	96.0	149	0.97 (0.92-0.99)	
Wash and Disinfect (*E. coli*)				
Yes	96.2	80	0.96 (0.86-0.99)	0.814
No	97.1	136	0.97 (0.91-0.99)	
SD >2 m^2 (*E. coli*)				
Yes	98.2	109	0.97 (0.90-0.99)	0.723
No	0.95.6	91	0.96 (0.86-0.99)	
Cleanease score (*E. coli*)				
≤2	98.2	169	0.97 (0.90-0.99)	0.534
>2	93.3	60	0.98 (0.87-1.00)	

TVC = Total Viable Count TCC = Total Coliform Count

Supplementary Table S3. Risk factors for feeding equipment that were not associated with the likelihood of meeting hygiene indicator targets

Variable (Hygiene Indicator)	% within target	Total number	Probability* (LCI-UCI**)	p-value
Milk Feeders				
Feeder Type (TVC)				
Single	31.3	118	0.33 (0.23-0.46)	0.670
Group	33.3	63	0.30 (0.17-0.46)	
Clean Area (TVC)				
Yes	36.7	60	0.38 (0.22-0.58)	0.394
No	29.7	121	0.29 ().18-0.42)	
Dry Area (TVC)				
Yes	40.6	32	0.42 (0.20-0.67)	0.389
No	30.2	149	0.30 (0.20-0.42)	
Cleaned with hot water and chemical (TVC)				
Yes	28.0	50	0.28 (0.13-0.50)	0.678
No	33.1	127	0.33 (0.22-0.46)	
Cleaned with hot water (TVC)				

Yes	28.6	91	0.29 (0.17-0.45)	0.641
No	0.34.9	86	0.34 (0.21-0.51)	
Cleaned with chemical (TVC)				
Yes	25.0	76	0.26 (0.14-0.43)	0.333
No	36.6	101	0.27 (0.17-0.40)	
Cleaned with cold water (TVC)				
Yes	43.9	57	0.42 (0.24-0.63)	0.184
No	25.8	120	0.27 (0.17-0.40)	
Cleaned at least daily (TVC)				
Yes	37.7	77	0.37 (0.23-0.53)	0.367
No	27.7	94	0.27 (0.16-0.42)	
Cleaned with hot water after every feed (TVC)				
Yes	53.3	30	0.50 (0.25-0.75)	0.115
No	32.1	137	0.27 (0.18-0.39)	
Cleaned with hot water at least daily (TVC)				
Yes	43.2	37	0.41 (0.21-0.65)	0.346
No	28.5	130	0.29 (0.18-0.41)	
Cleaned with chemical at least daily (TVC)				
Yes	42.1	19	0.37 (0.12-0.72)	0.714
No	30.4	148	0.31 (0.21-0.43)	
High TVC in mixing utensils (TVC)				
Yes	41.7	57	0.40 (0.22-0.61)	0.456
No	29.4	85	0.31 (0.18-0.47)	
Feeder Type (TCC)				
Single	51.7	120	0.52 (0.41-0.63)	0.424
Group	58.7	63	0.59 (0.45-0.72)	
Clean Area (TCC)				
Yes	55.0	60	0.56 (0.41-0.71)	0.759
No	54.5	123	0.53 (0.42-0.64)	
Dry Area (TCC)				
Yes	53.1	32	0.55 (0.34-0.74)	0.989
No	55.0	151	0.54 (0.44-0.64)	
Cleaned with hot water and chemical (TCC)				
Yes	48.1	52	0.49 (0.32-0.66)	0.507
No	56.7	127	0.56 (0.56-0.66)	
Cleaned with hot water (TCC)				
Yes	48.4	93	0.49 (0.36-0.62)	0.260
No	60.5	86	0.60 (0.46-0.72)	
Cleaned with chemical (TCC)				
Yes	56.4	78	0.58 (0.43-0.71)	0.504
No	52.5	101	0.51 (0.39-0.63)	
Cleaned with cold water (TCC)				
Yes	54.4	57	0.52 (0.36-0.68)	0.797
No	54.1	122	0.55 (0.44-0.66)	
Cleaned at least daily (TCC)				
Yes	57.1	77	0.56 (0.42-0.69)	0.706

No	52.1	96	0.52 (0.39-0.65)	
Cleaned with hot water after every feed (TCC)				
Yes	63.3	30	0.62 (0.39-0.80)	0.446
No	51.8	139	0.52 (0.41-0.62)	
Cleaned with hot water at least daily (TCC)				
Yes	56.8	37	0.55 (0.35-0.74)	0.852
No	53.0	132	0.53 (0.42-0.64)	
High TVC in mixing utensils (TCC)				
Yes	53.4	73	0.54 (0.38-0.68)	0.953
No	53.5	71	0.53 (0.38-0.68)	
Feeder Type (*E. coli*)				
Single	92.5	120	0.93 (0.86-0.96)	0.343
Group	88.9	63	0.88 (0.76-0.94)	
Clean Area (*E. coli*)				
Yes	95.0	60	0.95 (0.85-0.98)	0.232
No	89.4	123	0.89 (0.81-0.94)	
Dry Area (*E. coli*)				
Yes	96.9	32	0.97 (0.80-1.00)	0.251
No	90.1	151	0.90 ().83-0.94)	
Cleaned with hot water and chemical (*E. coli*)				
Yes	90.4	52	0.90 (0.77-0.96)	0.882
No	91.3	127	0.91 (0.84-0.95)	
Cleaned with hot water (*E. coli*)				
Yes	90.3	93	0.90 (0.81-0.95)	0.728
No	91.9	86	0.92 (0.83-0.93)	
Cleaned with chemical (*E. coli*)				
Yes	92.3	78	0.92 (0.83-0.97)	0.615
No	90.1	101	0.90 (0.81-0.95)	
Cleaned with cold water (*E. coli*)				
Yes	89.5	57	0.89 (0.77-0.96)	0.653
No	91.8	122	0.92 (0.84-0.96)	
Cleaned after every feed (*E. coli*)				
Yes	92.8	55	0.93 (0.81-0.97)	0.702
No	90.7	118	0.91 (0.83-0.95)	
Cleaned at least daily (*E. coli*)				
Yes	89.6	77	0.90 (0.79-0.95)	0.556
No	92.7	96	0.93 (0.84-0.97)	
Cleaned with hot water after every feed (*E. coli*)				
Yes	93.4	30	0.93 (0.75-0.98)	0.692
No	90.6	139	0.91 (0.84-0.95)	
Cleaned with hot water at least daily (*E. coli*)				
Yes	89.2	37	0.89 (0.71-0.96)	0.621
No	91.7	132	0.92 (0.85-0.96)	
Cleaned with chemical at least daily (*E. coli*)				

Yes	94.7	19	0.95 (0.68-0.99)	0.602
No	90.7	150	0.91 (0.84-0.95)	
High TVC in mixing utensils (*E. coli*)				
Yes	89.0	73	0.89 (0.79-0.94)	0.267
No	94.4	71	0.94 (0.86-0.98)	
High TCC in mixing utensils (*E. coli*)				
Yes	91.6	95	0.92 (0.84-0.96)	0.977
No	91.8	49	0.92 (0.80-0.97)	
Teats				
Pen Type (TVC)				
Single	59.6	47	0.60 (0.44-0.74)	0.796
Group	62.3	61	0.48 (0.48-0.75)	
Teat Type (TVC)				
Single	59.6	47	0.60 (0.43-0.74)	0.965
Group	61.5	26	0.62 (0.41-0.79)	
AMF	62.9	35	0.63 (0.44-0.79)	
Clean Area (TVC)				
Yes	65.1	43	0.65 (0.47-0.79)	0.590
No	58.5	65	0.59 (0.45-0.71)	
Dry Area (TVC)				
Yes	69.2	26	0.69 (0.46-0.85)	0.419
No	58.5	82	0.59 (0.46-0.70)	
Cleaned with hot water and chemical (TVC)				
Yes	64.7	34	0.64 (0.45-0.80)	0.69
No	59.5	74	0.60 (0.47-0.72)	
Cleaned with hot water (TVC)				
Yes	61.7	47	0.61 (0.44-0.75)	0.952
No	63.9	61	0.61 (0.47-0.74)	
Cleaned with chemical (TVC)				
Yes	60.0	45	0.59 (0.42-0.74)	0.775
No	61.9	63	0.62 (0.48-0.75)	
Cleaned with cold water (TVC)				
Yes	67.9	28	0.68 (0.47-0.84)	0.412
No	58.7	80	0.59 (0.46-0.70)	
Cleaned after every feed (TVC)				
Yes	62.1	29	0.61 (0.41-0.78)	0.943
No	60.8	79	0.61 (0.49-0.73)	
Cleaned at least daily (TVC)				
Yes	56.9	51	0.57 (0.42-0.71)	0.455
No	64.9	57	0.65 (0.50-0.78)	
Cleaned with hot water after every feed (TVC)				
Yes	54.6	11	0.53 (0.24-0.80)	0.599
No	61.9	97	0.62 (0.51-0.72)	
Cleaned with hot water at least daily (TVC)				
Yes	50.0	22	0.50 (0.29-0.72)	0.295
No	64.0	86	0.64 (0.52-0.75)	

Cleaned with chemical at least daily (TVC)				
Yes	40.0	15	0.40 (0.18-0.68)	0.131
No	64.5	93	0.65 (0.53-0.75)	
High TVC in mixing utensils (TVC)				
Yes	78.6	28	0.65 (0.38-0.85)	0.417
No	39.5	43	0.52 (0.37-0.67)	
High TCC in mixing utensils (TVC)				
Yes	58.9	56	0.59 (0.44-0.73)	0.288
No	40.0	15	0.42 (0.18-0.69)	
Pen Type (TCC)				
Single	62.5	48	0.65 (0.47-0.80)	0.338
Group	79	62	0.77 (0.62-0.88)	
Teat Type (TCC)				
Single	62.5	48	0.65 (0.47-0.80)	0.500
Group	80.8	26	0.79 (0.55-0.92)	
AMF	77.8	36	0.76 (0.55-0.90)	
Clean Area (TCC)				
Yes	71.1	45	0.72 (0.51-0.87)	0.923
No	72.3	65	0.71 (0.55-0.84)	
Dry Area (TCC)				
Yes	84.6	26	0.85 (0.59-0.95)	0.214
No	67.9	84	0.68 (0.54-0.80)	
Cleaned with hot water (TCC)				
Yes	77.1	48	0.75 (0.56-0.88)	0.597
No	67.7	62	0.69 (0.52-0.82)	
Cleaned with chemical (TCC)				
Yes	82.3	45	0.81 (0.62-0.91)	0.199
No	64.6	65	0.66 (0.50-0.79)	
Cleaned with cold water (TCC)				
Yes	69	29	0.71 (0.48-0.87)	0.959
No	72.8	81	0.72 (0.57-0.83)	
Cleaned after every feed (TCC)				
Yes	70	30	0.69 (0.46-0.85)	0.718
No	72.5	80	0.73 (0.58-0.84)	
Cleaned at least daily (TCC)				
Yes	67.9	53	0.68 (0.50-0.82)	0.518
No	75.4	57	0.75 (0.58-0.87)	
Cleaned with hot water after every feed (TCC)				
Yes	50	12	0.56 (0.24-0.83)	0.280
No	74.5	98	0.74 (0.61-0.84)	
Cleaned with hot water at least daily (TCC)				
Yes	60.9	23	0.62 (0.35-0.83)	0.337
No	74.7	87	0.75 (0.61-0.85)	
Cleaned with chemical at least daily (TCC)				
Yes	83.3	15	0.77 (0.42-0.94)	0.687

No	70.5	95	0.71 (0.57-0.82)	
High TVC in mixing utensils (TCC)				
Yes	70	30	0.73 (0.47-0.89)	0.745
No	67.4	43	0.68 (0.46-0.84)	
High TCC in mixing utensils (TCC)				
Yes	75.9	58	0.77 (0.59-0.89)	0.12
No	40	15	0.46 (0.17-0.78)	
Pen Type (*E. coli*)				
Single	83.3	48	0.83 (0.64-0.94)	0.821
Group	85.5	62	0.85 (0.69-0.94)	
Teat Type (*E. coli*)				
Single	83.3	48	0.84 (0.64-0.94)	0.933
Group	84.6	26	0.83 (0.56-0.95)	
AMF	86.1	36	0.87 (0.64-0.96)	
Clean Area (*E. coli*)				
Yes	84.4	45	0.87 (0.64-0.96)	0.707
No	84.6	65	0.83 (0.65-0.93)	
Dry Area (*E. coli*)				
Yes	96.2	26	0.96 (0.71-1.00)	0.161
No	81	84	0.81 (0.66-0.91)	
Cleaned with hot water and chemical (*E. coli*)				
Yes	91.2	34	0.91 (0.68-0.98)	0.41
No	81.6	76	0.82 (0.67-0.92)	
Cleaned with hot water (*E. coli*)				
Yes	81.2	48	0.83 (0.59-0.93)	0.562
No	87.1	62	0.87 (0.71-0.95)	
Cleaned with chemical (*E. coli*)				
Yes	88.9	45	0.89 (0.69-0.96)	0.505
No	81.5	65	0.82 (0.65-0.92)	
Cleaned with cold water (*E. coli*)				
Yes	79.3	29	0.81 (0.54-0.94)	0.593
No	86.4	81	0.86 (0.72-0.94)	
Cleaned after every feed (*E. coli*)				
Yes	80	30	0.81 (0.56-0.93)	0.575
No	86.2	80	0.86 (0.72-0.94)	
Cleaned at least daily (*E. coli*)				
Yes	77.4	53	0.78 (0.59-0.90)	0.169
No	81.5	57	0.91 (0.76-0.97)	
Cleaned with hot water after every feed (*E. coli*)				
Yes	58.3	12	0.71 (0.32-0.93)	0.266
No	87.8	98	0.87 (0.75-0.94)	
Cleaned with hot water at least daily (*E. coli*)				
Yes	65.2	23	0.71 (0.40-0.90)	0.125
No	89.7	87	0.77 (0.77-0.95)	
Cleaned with chemical at least daily (*E. coli*)				

Yes	86.7	15	0.87 (0.44-0.98)	0.877
No	84.2	95	0.84 (0.70-0.92)	
High TVC in mixing utensils (*E. coli*)				
Yes	76.7	30	0.82 (0.51-0.95)	0.701
No	88.4	43	0.87 (0.64-0.96)	
High TCC in mixing utensils (*E. coli*)				
Yes	82.8	58	0.85 (0.65-0.95)	0.996
No	86.7	15	0.85 (0.40-0.98)	
Mixing Utensils				
Mixing Utensil Type (TVC)				
Mix container	60.5	76	0.60 (0.47-0.73)	0.664
Jug	55.6	27	0.54 (0.33-0.74)	
Whisk	50	28	0.50 (0.30-0.71)	
Clean Area (TVC)				
Yes	56.9	51	0.58 (0.40-0.74)	0.933
No	57.5	80	0.57 (0.42-0.70)	
Dry Area (TVC)				
Yes	50	32	0.52 (0.30-0.73)	0.625
No	59.6	99	0.59 (0.46-0.71)	
Mixing Utensil Type (TCC)				
Mix container	84.2	76	0.82 (0.69-0.90)	0.956
Jug	79.3	29	0.79 (0.57-0.92)	
Whisk	75	28	0.80 (0.57-0.92)	
Clean Area (TCC)				
Yes	84.6	52	0.85 (0.67-0.94)	0.489
No	79	81	0.78 (0.62-0.88)	
Dry Area (TCC)				
Yes	78.8	33	0.81 (0.55-0.93)	0.975
No	82	100	0.81 (0.68-0.89)	
Mixing Utensil Type (*E. coli*)				
Mix container	98.7	76	0.98 (0.87-1.00)	0.466
Jug	96.6	29	0.97 (0.69-1.00)	
Whisk	100	28	1.00 (0.76-1.00)	
Clean Area (*E. coli*)				
Yes	100	52	1.00 (0.00-1.00)	0.603
No	97.5	81	0.98 (0.86-1.00)	
Dry Area (*E. coli*)				
Yes	100	33	1.00 (0.00-1.00)	0.709
No	98	100	0.98 (0.89-1.00)	

TVC = Total Viable Count TCC = Total Coliform Count
